# Opportunities and challenges in delivering remote primary care during the Coronavirus outbreak

**DOI:** 10.1186/s12875-022-01750-7

**Published:** 2022-05-31

**Authors:** V Kaufman-Shriqui, M Shani, M Boaz, A Lahad, S Vinker, R Birk

**Affiliations:** 1 Department of Nutrition Sciences, School of Health Sciences, Ariel University, Kiryat H’amada 3, 4070000 Ariel, Israel; 2 Department of Family Medicine, Central District, Clalit Health Services, Tel-Aviv, Israel; 3 Department of Family Medicine, Sakler School of Medicine, Tel Aviv University, Tel Aviv, Israel; 4 Braun School of Public Health & Community Medicine, The Hebrew University- Hadassah, Jerusalem, Israel; 5 Clalit Health Services, Jerusalem, Israel; 6 Leumit Health Services, Tel-Aviv, Israel; 7 Department of Family Medicine, Sackler Faculty of Medicine, Tel-Aviv University, Tel-Aviv, Israel

**Keywords:** Coronavirus, COVID-19, Health survey, Primary care, Telehealth, Telemedicine

## Abstract

**Background:**

Social distancing and lockdowns were implemented during the first period of the COVID-19 pandemic. Primary care physicians needed to adapt quickly to deliver remote care/telemedicine.

**Methods:**

A cross-sectional, 47-item online Google Survey was distributed through the Israel Association of Family Physicians (IAFP) mailing list between March 31-May 5, 2020. The questionnaire included demographics, physician characteristics, and information on usage and perceived telemedicine quality. Sampling weights by sex and age groups were applied.

**Results:**

One hundred fifty-nine primary care physicians (10.6% of registered IAFP members; 63.5% women; mean age 53.4 ± 10.4 years and median professional experience 21.3 years) replied to the survey. The majority (59.7%) of the participants performed a mixture of in-person along with phone counseling. About 40% had no former telemedicine experience. The majority indicated that telephone and video formats were inferior to in-person consultation (68%, 57.1% online and phone, respectively). The overall counseling quality grade (on a 1–10 scale,)median (IQR)) was 6.2 (3) for telephone and 7(2) for video. While 66.9% reported experiencing no challenges, 10% had technical problems, 10% interpersonal problems, 5.6% scheduling difficulties, and 7.5% other difficulties. Majority of 56.6% physicians indicated they prescribed more antibiotics,16.4% sent more blood tests, 24.5% referred more to experts, and 49.7% referred more to imaging in comparison to usual counseling. Higher phone quality score was significantly associated with physicians who indicated not prescribing more antibiotics during the pandemic (OR = 0.30, 95%CI 0.134–0.688, *p* = 0.004).

Higher online quality score was associated with physicians who indicated not sending more blood tests during the pandemic (OR = 0.06 95%CI 0.008–0.378, *P* = 0.003).

**Conclusions:**

Our findings suggest telehealth holds considerable promise for counseling in the primary care setting. However, interpersonal challenges raised by physicians should be understood in-depth to develop tailored training and further examine it in randomized trials while integrating patient-reported outcomes. Finally, further research on utility, cost, and cost-efficiency during remote counseling with follow-ups, medical prescribing, and additional referrals is needed.

## Introduction

Primary care is characterized by long-term patient-physician relationships and continuity. The introduction of telemedicine and the ability to communicate without in-person clinic visits has created new modalities for interaction with patients. Telemedicine has been found to be effective and to improve clinical outcomes in a variety of medical conditions, including wound care, psychiatry, and chronic conditions like diabetes mellitus and hypertension [1]. Informational videos providing instruction in self-monitoring and health education and awareness tailored to specific morbidities have been shown to reduce hospitalizations among people at high risk for re-hospitalization with heart failure, chronic obstructive pulmonary disease (COPD), diabetes mellitus, or hypertension [2]. Additionally, at a large hospital outpatient setting staffed by physicians trained to deliver telemedicine, both patients and clinicians found no difference in the quality of virtual vs. in-person office visits [3].

Video consultations were rated as positive experiences, especially among working people or patients with mobility problems [4]. In a system allowing patient access to medical care 24 h a day via web and phone-enabled virtual visits, 85% of patients rated their satisfaction with their telemedicine physician at the highest rank [5]. High satisfaction rates were also obtained by patients with celiac who used telemedicine during the COVID-19 pandemic [6]. However, according to a national survey, fewer than 10% of family physicians in the USA provided e-visits, despite patient satisfaction [7]. Common barriers to telemedicine use included technical challenges, resistance to change, as well as patient age and level of education [8]. Physicians' important concerns working in a pediatric telemedicine service were difficulties diagnosing from a distance and treating unfamiliar patients [9]. A recent Cochrane review identified gaps in knowledge of physicians’ perspective, experiences, adherence, and satisfaction in delivering medical care using mobile technologies [10].

The COVID-19 pandemic has forced rapid adjustment for family physicians to practice telemedicine. The general lockdown coupled with patient anxiety about clinic visits has forced family physicians to adopt telemedicine and distance communication with patients instead of in-person encounters. A recent study conducted among primary care physicians in Israel indicated that daily use of WhatsApp in professional practice reduced the need for in-person counseling [11].

In the current study, we aimed to evaluate physician experiences and attitudes to the change in practice paradigm during the COVID-19 pandemic.

## Methods

### Overall study design and plan

A cross-sectional survey was conducted using online platform. We used a convenience sample of primary care physicians replied to an online survey distributed via e-mail by the Israeli Association of Family Physicians to all members. The study used a Google Survey platform. Data collection was performed from March 31-May 5, a period of time during which the Israeli population was under wide-scale lockdown.

### Ethics approval and consent to participate

The study was approved by the Institutional Ethics Committee of Ariel University. Each participant provided electronic written informed consent prior to responding to the survey.

### Survey tool

The survey included 47 questions, including sociodemographic information (6 items), characteristics of routine primary care setting and employment (8 items), characteristics of primary care setting during the outbreak (4 items), features experiences and quality of telemedicine including phone or video or a combination of them (8 questions on phone counseling and additional 12 questions on video counseling), physician experiences (5 items), and perceived patient experiences (4 items). Some questions were modified from a telemedicine questionnaire for healthcare workers [12, 13]. The questions selected requested information on overall quality, technical quality, clinical quality, and organizational difficulties (on a scale of 1–10) and additional questions on technical difficulties and future use (on a scale of 1–4). Survey participants were also asked to indicate their work setting, the number of registered patients, the weekly number of work hours, if and how they continued to work during the COVID-19 outbreak, and the proportion of patients with whom they corresponded using e-mail, WhatsApp, or phone prior to the outbreak. Additionally, physicians were asked whether non-face-to-face meetings were an option and to estimate the percentage of their patients who were invited to the clinic following a phone or video consultation (five categories: never, 25%, 50%, 75%, all). Respondents were asked to rate the quality of remote platforms to in-person counseling. Participants were requested to describe the challenges that they experienced during counseling sessions. This information was thematically analyzed by two researchers who reached a consensus on the main topics. Additionally, participants used a 10-point Likert scale to estimate client comfort level during remote counseling. Participants replied to the survey in Hebrew. Prior to publishing the survey online, the survey questions and usability of the online platform were referred to a focus group of 10 potential participants, who were asked to assess readability and clarity. Based on their feedback, minor amendments were made. The final questionnaire was reviewed by ten expert primary care physicians providing expert validity.

### Comparison between the study population and the general population of primary care professionals in Israel

To determine the representativeness of the study sample, participant age by sex distributions were compared to those of the target population (Israeli primary care physicians), using the most recent data available [14]. As shown in Table 1, the survey population was younger and included a greater proportion of women than the target population. Age distribution differed significantly by sex, such that the study sample included a greater proportion of both men and women between the ages 41–60, while the target population was characterized by a greater proportion of men between the ages of 51–70 years, and women between the ages of 41–70 years (χ^2^ = 225.29, DF = 5, *p* < 0.001). Sample weights were created based on the national age and sex distribution and applied to the survey population.

**Table 1:** Comparison between the distribution of family physicians by sex and age group in survey and the country^a^

Age group	Men in the survey n,(%)	Men in the profession in the country (n,%)	Women in the survey (n,%)	Women in the profession in the country (n,%)
Lower than 30	0 (0)	75 (2.7)	1(1.0)	27 (1.2)
31–40	8 (14.0)	336 (12.0)	22(21.8)	297 (13.2)
41–50	20 (35.1)	541 (19.5)	39 (38.6)	573 (25.5)
51–60	14 (24.6)	809 (29.1)	29(28.7)	746 (33.2)
61–70	10 (17.5)	870 (31.3)	10 (9.9)	527 (23.5)
71 and above	5 (8.8)	130 (4.7)	0 (0)	59 (2.6)
Missing		20 (0.7)		15 (0.7)
Total	57	2781	101	2244

^a^Data on the distribution of physicians in the country were taken from Ginat A. Practitioners and specialists in family medicine in Israel, 2018. Jerusalem, Israel: Administration of Strategic and Economic Planning; 2019

### Data analysis

SPSS v. 25.0 (IBM Inc., USA) was used for all statistical analyses. First, the weighted characteristics of study population were described., Distributions of continuous variables were assessed for normality using the Kolmogorov–Smirnov test. Normally distributed continuous variables are described using mean ± standard deviation. Continuous data with distributions significantly deviating from normal are described as median (interquartile range). Categorical variables such as the percentage of participants providing a given response were described using frequency counts and expressed as n (%). Associations between sociodemographic and occupational characteristics of physicians and the overall quality of phone and online counseling were examined using survey-weighted multiple logistic regression analyses with stepwise variable selection. The independent variables associated with the overall quality variable at a significance level of *p* ≤ 0.10 were considered for inclusion in multivariate models. Two separate models were developed, and the median value of the quality score at each type of consultation was assigned as the cutoff value. All tests are two-sided and considered significant at *p* < 0.05.

## Results

### The survey participants

Of 1,500 registered members of the Israel Association of Family Physicians (IAFP), 159 replied to the survey (10.6%). Table 2 describes the characteristics of survey participants. The mean participant age was 53.4 ± 10.4 years and most were female. The majority (90%) of the participants were specialists in family medicine, and 60% were employees of one of the national health maintenance organizations (HMO), while 30.8% were independent physicians, and 8.8% worked in both employment settings. Median professional experience was 20.0 years. The majority of physicians (59.8%) reported more than 1,000 patients in their clinics. Most participants (73.6%) indicated that during routine times, 50% of their patients or more would require a physical examination. A greater proportion of the physicians indicated that the most prevalent communication type with patients prior to the Covid-19 outbreak was e-mail correspondences, followed by phone calls and, to a lesser extent, WhatsApp messages.

**Table 2:** Characteristics of survey participants

Characteristic	Result *n* = 159
***Age, years (Mean* ± *SD)***	53.4 ± 10.4
***Sex n, (% female)***	101.0 (63.5) Weighted: 49.9%
***Specialization in medicine n (%)***	
Family medicine	143.0 (89.9)
Intern in family medicine	9.0 (5.7)
Practicing family medicine with no specialization	4.0 (2.5)
Other medical specialization	3.0 (1.9)
***Years of professional experience Median (IQR)***	21.3 (17.0)
***The main form of employment n (%)***	
Employee	96.0 (60.4)
Independent physician	49.0 (30.8)
Both employee and independent physician	14.0 (8.8)
***How many registered patients do you have? n(%)***	
More than 1500	45.0 (28.3)
Between 1000–1500	50.0 (31.5)
Between 500–1000	46.0 (28.9)
Less than 500	18.0 (11.3)
***Number of hours/week performing counseling (prior to the COVID-19 outbreak) (Mean* ± *SD)***	27.9 ± 10.7
***At routine times, which proportion of your patients’ visits require a physical examination n (%)***	
All patients	4.0 (2.5)
75% of the patients	24.0 (15.1)
50% of the patients	89.0 (56.0)
25% of the patients or less	42.0 (26.4)
***Before the Corona crisis, did you give your cell phone number to patients routinely? n(%)***	
*Yes*	29.0 (18.2)
***Before the Corona crisis, did you routinely hold WhatsApp correspondence with patients? n(%)***	
Yes	25.0 (15.7)
***Before the Corona crisis, did you routinely communicate with patients *via* your e-mail? n(%)***	
Yes	65.0 (40.9)

*Abbreviations*: *IQR* Interquartile range

### Characteristics of primary care counseling during COVID-19 outbreak

Table 3 describes the characteristics of primary care counseling during the study period. The majority of physicians reported full-time employment (92.5%) but reported a decrease in meeting patients to 18.3 ± 12.3 h on average compared to 27.9 ± 10.7 mean hours prior to the pandemic. While 41.5% reported no change in client characteristics, 40.3% reported accepting younger clients, and 9.4% older clients.

**Table 3:** Characteristics of counseling since the COVID-19 outbreak^a^

Characteristic	Result *N* = 159
***Work capacity after the onset of the COVID-19 n (%)***	
Full-time	147 (92.5)
Part-time	12.0 (7.5)
***Number of hours/week performing in-patient counseling (since the COVID-19 outbreak) (Mean* ± *SD)***	18.3 ± 12.3
***Changes in patients characteristics during the COVID-19 outbreak***	
No changes in patients characteristics	66 (41.5)
Younger patients	64 (40.3)
Older patients	15 (9.4)
Other	14 (8.8)
***Was it possible for the patients to schedule a non-face-to-face meeting in your clinic?***	
***yes n,(%)***	147 (92.5)
***The setting of counseling after the COVID-19 outbreak***	
Usual counseling and phone counseling	95 (59.7)
Only phone counseling	8 (5.0)
Only online counseling	2 (1.3)
Only phone and online counseling	2 (1.3)
Usual counseling as well as phone and online counseling	52 (32.7)

^a^Calculated with the application of sample weights

During the pandemic, the majority of the physicians (92.5%) employed some form of telemedicine. Types of mixed counseling were frequent, with the majority of the participants who performed a mixture of usual face-to-face counseling with phone counseling (59.7%). The greatest percentage of respondents who performed online counseling used a mixture of video platforms. The most frequently used platform was WhatsApp (53.9%), with 22.2% who used it as the only option, followed by ZOOM (22.2%) and Microsoft Teams (16.6%). An additional 7.3% used Facebook Messenger, Skype, and Unicko.

Characteristics of primary care consultation over the phone and when using online video platforms are presented in Table 4.

**Table 4:** Characteristics of quality of telemedicine using the phone and online platforms^a^

Question/Response	Result
**Counseling over the phone *n(%)***	*N* = 157
***Previous experience with performing telephone counseling***	
Yes	51 (32.5)
Limited experience	50 (31.8)
No	56 (35.7)
***How would you compare the duration of phone counseling in comparison to face-to-face counseling?***	
Similar in duration to face-to-face counseling	33 (21.0)
Much longer than face-to-face counseling	15 (9.6)
Slightly longer face-to-face counseling	17 (10.8)
Much shorter than face-to-face counseling	23 (14.6)
Slightly shorter than face-to-face counseling	69 (44.0)
***Percentage of the patients that were requested to arrive at a face-to-face meeting following a telephone counseling***	
None	4 (2.5)
25%	141 (89.9)
50% and above	12 (7.6)
***Percent of patients who shared photographs or videos during the meeting [median (interquartile range)]***^a^	4 (5.0)
***How would you compare phone counseling to usual counseling (face-to-face)? n (%)***	
Superior to face-to-face counseling	12 (7.6)
Similar to face-to-face counseling	30 (19.1)
Inferior to face-to-face counseling	108 (68.8)
Not certain	7 (4.5)
***The overall quality of counseling [median (interquartile range)]***^***b***^	6.2 (3.0)
***Level of physician’s convenience in performing phone counseling n,(%)***	
Highly convenience	34 (21.7)
Reasonably convenience	65(41.4)
Not highly convenient	48 (30.6)
Not convenient at all	10 (6.3)
***Intention to use telephone counseling in the future n (%)***	
Yes	41(26.2)
Yes, while combining phone and face-to-face counseling	85 (54.2)
Not certain	23 (14.6)
No	8 (5.0)
**Counseling using an online (video) platform**	
***Previous experience with performing online video counseling n (%)***	*N* = 56
Yes	22 (39.3)
Limited experience	11(19.6)
No	23 (41.1)
***How would you compare the duration of phone counseling in comparison to face-to-face counseling? n (%)***	
Similar in duration to face-to-face counseling	18 (32.1)
Much longer than face-to-face counseling	2 (3.6)
Slightly longer face-to-face counseling	11(19.7)
Much shorter than face-to-face counseling	7 (12.5)
Slightly shorter than face-to-face counseling	18 (32.1)
***Percentage of the patients that were requested to arrive at a face-to-face meeting following a telephone counseling n (%)***	
None	12 (21.4)
25%	42 (75.0)
50% and above	2 (3.6)
***How would you compare online counseling to usual counseling (face-to-face)? n (%)***	
Superior to face-to-face counseling	2 (3.6)
Similar to face-to-face counseling	20 (35.7)
Inferior to face-to-face counseling	32 (57.1)
Not certain	2 (3.6)
***Level of physician’s convenience in performing video counseling n,(%)***	
Highly convenience	18 (32.1)
Reasonably convenience	23 (41.1)
Not highly convenient	14 (25.0)
Not convenient at all	1 (1.8)
***Intention to use telephone counseling in the future n (%)***	
Yes	30 (53.5)
Yes, while combining phone and face-to-face counseling	15 (26.8)
Not certain	9 (16.1)
No	2 (3.6)
***Quality of counseling [median (interquartile range)]***^a^	
Overall quality^b^	7 (2)
Technical quality^b^	6 (2)
***Physician challenges in performing the online counseling***	
Organizational difficulties^b^	7 (3)
Technical difficulties, n (%)	16 (10.0)
Difficulties due to lack of physical examination, n (%)	16 (10.0)
Scheduling difficulties, n (%)	9 (5.6)
Other ^c^, n (%)	12 (7.5)
***In comparison to the face-to-face meeting, have you prescribed more antibiotics? n (%)***	*N* = 159
Yes	90 (56.6)
Prescribed as usual	69 (43.4)
***In comparison to the face-to-face meeting, have you sent more blood tests? n (%)***	
Yes	26 (16.4)
Blood tests as usual	133 (83.6)
***In comparison to the face-to-face meeting, have you referred patients to experts more than usual? n (%)***	
Yes	39 (24.5)
Referrals as usual	120 (75.5)
***In comparison to the face-to-face meeting, have you referred to more imaging tests? n (%)***	
Yes	79 (49.7)
Imagining as usual	80 (50.3)
***Patient Outcomes***	*N* = 159
***Perceived level of patient comfort in receiving remote counseling (phone or video) [median (interquartile range])***	8 (3)
***Types of difficulties reported by patients during online (video) or phone counseling n (%)***	
Technical difficulties	48 (30.0)
Interpersonal communication difficulties	33 (20.6)
Difficulties due to lack of physical examination	22 (13.8)
Difficulties/inconvenience from conducting the session in the home environment	7 (4.4)
Other^c^	14 (8.8)

^a^Open scale, replies were numerically restricted to 0–100 percent

^b^Items scored on a 10-point Likert scale ranging from 1–10, where 1 = “very low” to 10 “very high”

^c^Other difficulties included themes of trust, age difficulties, hearing and sight disability which prevented patients from communicating well using the suggested telemedicine, and a desire to see the physician in person

^d^Calculated with the application of sample weights

### Consultation over the phone

More than one-third of respondents (35.7%) indicated they had no previous experience performing consultation over the phone prior to the COVID-19 pandemic, while another third (32.5%) had previous experience. The majority (58.6%) of respondents indicated phone counseling was of shorter duration than in-person visits. Of these, 14.6% indicated that consultation was significantly shorter than in-person consultations. Most respondents (89.9%) indicated that following phone counseling, they needed to invite about 25% of the patients to the clinic for an in-person visit. Only 4% (5 IQR) indicated that patients shared videos or photos with them during phone counseling. The majority of respondents (68.8%) indicated that phone consultation was inferior to in-person visits. On a scale of 1–10, the median (IQR) rate for the overall quality of phone counseling was 6.2 (3.0). Many respondents (62.1%) thought phone counseling was reasonably or highly convenient. The majority (80.4%) of respondents indicated they intend to use phone counseling in the future and only 5% indicated they would not use it.

### Consultation via online (video) platforms

Many (41.1%) of respondents indicated they had no previous experience performing consultation using online platforms prior to the COVID-19 pandemic, while 39.3% reported having had previous experience. The majority (64.1%) of respondents indicated online counseling was either similar in length or of shorter duration than in-person visits. Most respondents (75.0%) indicated that following online counseling, they needed to invite about 25% of the patients to the clinic. The majority of respondents (57.1%) indicated online consultation was inferior to in-person visits. On the other hand, most respondents (73.2%) thought online counseling was reasonably or highly convenient, and only 1.8% thought it was not convenient. The majority (80.3%) of respondents indicated they intend to use online counseling in the future.

### Challenges and qualities of remote counseling

On a scale of 1–10, the median (IQR) rate for the overall quality of online counseling was 7 (2), the technical quality 6 (2), and for organizational difficulties, 7 (3). Physicians reported a variety of challenges in performing online consultations. While 66.9% reported experiencing no challenges, 10% had technical problems, 10% interpersonal problems, 5.6% scheduling difficulties, and 7.5% other difficulties. In reply to questions addressing characteristics of the medical treatment, in any remote platform (either phone or online), 56.1% indicated they prescribed more antibiotics, 16.4% sent more patients than usual to perform blood tests, 24.5% indicated an increase in referral to expert physicians and 49.7% indicated an increase in referral to imaging. Main themes which emerged from an open-ended question that requested further information on challenges identified four populations with which telemedicine was challenging in particular: older people; low socioeconomic status populations; people with hearing disability; and people with low technological literacy.

In reply to the question, “What are your primary needs to perform a higher quality remote?” the majority of physicians described a need for high-quality equipment (camera, headphones, adequate chair for long sitting); infrastructure (strong internet network resources, adequate light); and a better scheduling system for the patients with reminders to prevent missed visits. Additionally, due to the situation of working under lockdown when some physicians were also parents for young children, physicians highlighted the need for assistance with childcare while they are at work.

### Patient outcomes

Physician-reported patient outcomes included the patient’s level of comfort and difficulties as perceived by the participating physician. The median (IQR) level of patient comfort was 8 (3). Physicians reported that 46.3% of their patients reported no difficulties with telemedicine methods, but the rest reported at least one problem. The most frequently reported patient problems were technical difficulties (30.0%), interpersonal challenges (20.6%), lack of physical checkup and measurements (13.8%), problems due to holding the meeting in the patient’s home environment (4.4%), and 8.8% who reported on other difficulties.

### Associations between sociodemographic and occupational characteristics of primary care physicians and the overall quality of phone and online counseling

In a multivariate logistic regression in which the dependent variable was the total quality score of counseling using the phone greater than 6, the only significant predictor variable was prescribing more antibiotics. Specifically, respondents who reported prescribing more antibiotics had 3.03% lower odds for a higher quality score (OR = 0.303 95%CI 0.134–0.688, *p* = 0.004) (Table 5). In a multivariate logistic regression in which the dependent variable was the total quality score of counseling using an online video platform higher than a median value of 7, which included sex, age, and previous experience in video counseling, an increase in referrals to blood tests was the only significantly associated variable with a 6% reduction in odds of reporting a greater quality score (OR = 0.06 95%CI 0.008–0.378, *P* = 0.003) (Table 6).

**Table 5:** Factors associated with a higher overall quality score of phone counseling in a multivariable logistic regression analysis*^a^

	**Odds Ratios**	**95% CI**	***p*-value**
Age (years)	0.99	0.959–1.038	0.91
Sex (men vs. women)	1.02	0.468–2.204	0.968
Prescribing more antibiotics	0.30	0.134–0.688	0.004
Previous experience using a phone in primary care counseling (no experience vs. experience)	1.14	0.499–2.607	0.756
Constant	5.34		0.145

^a^Calculated with the application of sample weights

^*^Quality score was calculated as the sum of scores of the item questions on quality, technical quality, clinical quality, and organizational difficulties (on a scale of 1–10). Higher quality score was assigned a total quality score higher than the median value of 6 points for performing telemedicine using the phone

**Table 6:** Factors associated with a higher overall quality score of video counseling in a multivariable logistic regression analysis^a^**

	**Odds Ratios**	**95% CI**	***p*-value**
Age (years)	1.03	0.948–1.113	0.516
Sex	0.62	0.159–2.444	0.498
Referring patients to more blood tests	0.06	0.08–0.378	0.003
Previous experience using the video platforms in primary care counseling (no experience vs. experience)	1.51	0.329–6.956	0.59
Constant	0.84		0.933

^a^Calculated with the application of sample weights

^**^Quality score was calculated as the sum of scores of the item questions on quality, technical quality, clinical quality, and organizational difficulties (on a scale of 1–10). Higher quality score was assigned a total quality score higher than the median value of 7 points for performing telemedicine using the video platforms

## Discussion

In the present study, we described the unique characteristics of consultation during the first phase of the COVID-19 pandemic in Israel. During the outbreak, most primary care physicians reported a decrease in the in-patient visits, while most of them performed mixed counseling by phone, online platforms, or both methods; however, more than half of the respondents implemented telemedicine without previous experience or training in using these modalities. Physicians reported counseling using either phone or online platforms relatively shorter in duration than the usual care.

Telemedicine holds important potential advantages in primary care medicine including continuity of care, monitoring symptoms and behavior in real-time; further, it enables clinicians to reach out to populations who live remotely or are confined to bed [4, 15]. It has been associated with improved clinical outcomes in various acute conditions such as wound care and chronic diseases such as diabetes mellitus [1] and even follow-up after surgery [16]. Telemedicine has even been shown to be as effective as face-to-face care in managing heart failure obtaining glucose control among patients with diabetes [17]. During the COVID-19 pandemic, rapid adjustments for delivering telemedicine were reported in several medical fields, for example, neurology [18] and orthopedics [19], while in other fields, such as ophthalmology, only partial solutions were reported [20]. Overall, studies reported high satisfaction and high quality of telemedicine. Telephone and video counseling were rated highly by primary care physicians and patients [3, 4], with video counseling receiving superior ratings [21]. The physicians in our study indicated that delivering care using both telephone and video was reasonable to highly convenient and rated the overall quality of telephone and online platforms relatively high despite pointing it inferior to face-to-face counseling. The majority of the physicians stated they intend to continue using telemedicine options following the crisis.

Despite the substantial benefits of telemedicine, challenges in delivering telemedicine were frequently reported in the literature, including: clinical uncertainty, lack of technological solutions [9], cyber vulnerabilities, and regulation challenges [8, 16]. In our study, several challenges were highlighted by physicians concerning their ability to deliver optimal remote care. Physicians reported only moderate technical quality as well as serious organizational difficulties. In qualitative inputs, most physicians reported suboptimal technical quality of the internet or quality of pictures or audio. Similar technical problems were reported in other studies [8, 22, 23]. Additional reported barriers were the lack of physical examination and scheduling difficulties (e.g., patients missing appointments or hardship in scheduling appointments).

Remote care poses a challenge regarding resource utilization, as was found in our study. Compared to usual care, telemedicine practice resulted in increases in prescriptions written for antibiotics (56.6%), blood tests (16.4%), referrals to a specialist (24.5%), and referrals for imaging (49.7%). These findings are consistent with previous research among Israeli pediatricians, in which a 20% increase in prescription writing for antibiotics for suspected pneumonia or otitis media scenarios was reported [24]. Despite the overall trend of decrease in antibiotics usage during 2019–2020. During the first months of the outbreak, a slight increase in prescribing antibiotics was reported in England between March 2020 compared to March 2019 [25]. Only after, in May 2020, the WHO published guidelines for appropriate usage of antibiotics when managing COVID-19, and increased awareness followed by a decrease in antibiotic use was noted in the primary healthcare setting [25]. Interestingly, the only variable in our study that was significantly associated with a higher reported quality score (both telephone and video) was blood tests – specifically, physicians who did not increase the number of blood tests ordered during the pandemic compared to the usual care reported higher quality scores. Unlike fee-per-practices payment systems, the Israeli universal health care system does not grant or deny payment from physicians according to referrals or practices [26]. Thus, physicians who avoided submitting additional blood checks may be those with higher confidence in performing the counseling.

In our research, physicians indicated a 40.6% increase in clinic appointments among younger individuals. Performing counseling to older people, people with hearing disabilities, or lower technological literacy were less satisfactory. Those challenges align with those from a recent review that examined inequalities in the primary care setting. The review concluded that telephone consultations were used more by younger working-age people, the very old, and non-immigrants, with internet-based consultations more likely to be used by younger people [27]. An additional survey from Israel reported a positive association between younger age and higher eHealth literacy and satisfaction and usage of telemedicine [28].Efforts should be made to prevent inequalities in delivering primary care due to structural differences. Despite relatively high patient comfort in receiving remote care, some prevalent difficulties were reported. Technical difficulties were most prevalent, followed by interpersonal communication difficulties.

Our study emphasizes telemedicine’s potential benefits, allowing continuous medical care while ensuring the necessary patient and physician safety in light of possible crisis-induced lockdown. Our study adds knowledge on specific challenges to the Israeli healthcare and HMOs in performing remote counseling. It seems necessary that HMOs improve technical platforms and scheduling difficulties and invest in enhancing accessibility of telehealth to older people and patients with technological challenges, thus improving equitable healthcare. Specific training and care modalities should be developed to address interpersonal difficulties and the lack of physical examinations. Additional aspects such as professionalism and webside manners should be considered during training. Israeli healthcare should invest resources to examine alternatives to optimize remote counseling [29, 30] through randomized clinical trials and include patient-reported outcomes. Further research is needed to assess the costs and system resource allocation and examine whether the usage of medical resources was necessary or preventable. Economic analysis should compare the actual cost and cost-efficacy of extra resource usage reported by our sample during the pandemic to improve telehealth drove medical decision-making models. Additional benefits can be obtained from qualitative research by interviewing physicians’ about their experiences in delivering remote healthcare using different modalities.

The present study has several limitations. First, our data are cross-sectional, and as such, causality cannot be inferred. Second, since we used a convenience sample and received a 10.6% reply rate**,** the characteristics of the respondents do not represent the target population, and the generalizability of our findings may be limited. Specifically, the study population was younger and included a larger proportion of women than the target population. Despite the lack of representativeness, we have applied sample weights in all our statistical analyses. Our findings were stable and showed consistency between weighted and unweighted samples.

Additionally, we relied on physicians' estimates when referring to percentages of further referred patients following an online visit. Moreover, We did not collect data about the characteristics of the clinic population, so we could not analyze the differences between populations.

We thus conclude that the findings may be generalizable to the target population. It is possible that those who responded to the survey were those who are also more oriented to telemedicine platform usage. However, the unusual circumstances that forced all physicians to respond quickly to the lockdown and the timing of the survey may indicate that these are real-time responses. Our data collection was performed early on during the first phase of the COVID-19 pandemic. The healthcare response and the nature of the pandemic have changed since. In a recent survey conducted among primary care providers and patients, both reported high levels of satisfaction with telemedicine visits in a primary care setting. Both providers and patients reported a desire to see telemedicine visits continued after the pandemic. Benefits were highly associated with patients who needed to drive more than 30 min to the visit and when the technical quality was high [31]. Additional information from a web-based Israeli survey among patients emphasizes that while the majority of 80% of patients used telemedicine during the lockdown, men and people with chronic diseases obtained higher willingness to use telemedicine in the future [32]. These recent publications, and additional ones [33], indicate that a growing number of individuals in OECD countries are now readily served by telehealth systems [34]. It seems that the circumstances have encouraged more providers and patients to use telemedicine as part of their routines. Efforts should be made to improve quality and test modalities of care using this technology.

## Conclusions

Our findings suggest that telemedicine served as a reasonable method for delivering primary care consultations during a global pandemic. However, additional research is needed to establish appropriate standards of care and practice using telemedicine in both crisis and routine periods.

## Data Availability

The datasets generated during and analyzed during the current study are not publicly available due to lack of agreement of the medical organization who distributed the questionnaire. However, data will be available from the corresponding author if a specific reasonable request will be transferred.

## Abbreviations

COPD: Chronic obstructive pulmonary disease
COVID-19: Coronavirus Disease 2019
HMO: Health maintenance organizations
IQR: Intra quartile range
IAFP: Israel Association of Family Physicians
